# Molecular genetic identification of Wuzhishan ant chicken, a newly discovered resource in China

**DOI:** 10.3389/fvets.2024.1319854

**Published:** 2024-06-19

**Authors:** Lihong Gu, Fanghu Wu, Xinli Zheng, Xiaohui Zhang, Yanmin Chen, Lizhi Lu, Xiangxiang Liu, Shuhui Mo, Zhe Chao, Zhongchun He, Yuanyuan Shang, Dong Wei, Sheng Wei, Youyi Chen, Tieshan Xu

**Affiliations:** ^1^Institute of Animal Science and Veterinary Medicine, Hainan Academy of Agricultural Sciences, Haikou, China; ^2^College of Animal Science and Technology, Henan University of Science and Technology, Luoyang, China; ^3^Wuzhishan Animal Science and Veterinary Medicine and Fishery Service Center, Wuzhishan Agricultural and Rural Bureau, Wuzhishan, China; ^4^Institute of Animal Husbandry and Veterinary Science, Zhejiang Academy of Agricultural Sciences, Hangzhou, China; ^5^Wuzhishan Ant Chicken Cooperative, Wuzhishan, China; ^6^Tropical Crops Genetic Resources Institute, Chinese Academy of Tropical Agricultural Sciences, Haikou, China

**Keywords:** Wuzhishan ant chicken, genetic diversity, whole-genome resequencing, genetic structure, genetic resource

## Abstract

**Introduction:**

The Wuzhishan ant (MY) chicken exhibits significant differences from other chicken breeds. However, the molecular genetic relationship between the MY breed and other chicken breeds has not been assessed.

**Methods:**

Whole-genome resequencing was used to compare genetic diversity, nucleotide diversity, the fixation index, the linkage disequilibrium coefficient, and phylogenetic tree relationships between the MY breed and the Wenchang (WC), Danzhou (DZ), Bawangling (BW), and Longsheng Feng (LF) breeds.

**Results:**

A total of 21,586,378 singlenucleotide polymorphisms and 4,253,341 insertions/deletions were screened out among the five breeds. The MY breed had the second highest genomic genetic diversity and nucleotide diversity and the lowest LD coefficient among the five breeds. Moreover, the phylogenetic tree analysis showed that individual birds of each breed clustered together with those of their respective breeds.

**Discussion:**

Our data indicated that the MY breed is distinct from the other breeds and can be considered a new genetic resource.

## 1 Introduction

Hainan Province is located at the southernmost tip of China and has a tropical and subtropical climate. It is surrounded by the sea and has a forest coverage rate of 62.1%. Owing to its distinct geography, climate, and ecological conditions, indigenous Hainan livestock and poultry varieties have unique characteristics. The region has several local chicken breeds, including Wenchang (WC), Danzhou (DZ), Bawangling (BW), and Wuzhishan ant (MY) chickens. WC and DZ chickens have been listed in the “National Livestock and Poultry Genetic Resources Breed List” by the Chinese government (1).

Animal Genetic Resources (AnGRs) refer to the collective of animal species, breeds, and strains that are currently, or potentially, of significant economic, scientific, and cultural heritage value to humanity. In terms of conservation and utilization, breed diversity typically holds greater importance than species diversity. Two methods comprise phenotypic and molecular identification techniques for identifying individual members of a livestock breed. Traditionally, phenotypic identification has been used to identify the breed of an individual in livestock genetic diversity studies. The major drawback of this technique lies in the fact that it can only observe genetic diversity at the phenotypic level, which does not always correspond to the actual diversity at the DNA level. The molecular method for identifying breed characteristics involves two primary approaches, utilizing either protein markers or DNA markers (2). The two most widely utilized DNA techniques are the variable number of tandem repeats (VNTRs), which include minisatellite and microsatellite markers, and single-nucleotide polymorphism (SNP)-based techniques (3, 4). The VNTR and SNP techniques have a more widespread application than other molecular markers.

The MY breed was identified in Wuzhishan City, located in the central-southern mountainous region of Hainan Province, during the national livestock and poultry genetic resources census conducted in China in 2021. It is a small-sized breed with consistent physical characteristics known for its stable inheritance of key economic traits and is characterized by thin skin, tender meat, and delicate bones when cooked (5). MY chickens, known as “ant chickens” locally owing to their small size, are raised in the wild by local ethnic minorities. Roosters have a cockspur, and their plumage is fancy, featuring hemp and black hemp and date-red colors, with green belly feathers, gray or black feet, and white ear lobes. The hens have grayish-white and brownish-black hemp feathers, gray or black feet, and white ear lobes. Sisal and black sisal roosters have black or sisal, or brown and white interspersed primary and secondary wing feathers. Red-feathered roosters have date-red neck feathers and black or date-red primary and secondary wing feathers (Supplementary Figure S1). MYs have a relatively close relationship with the location, culture, and historical origins of the natural ecological environment. According to “Zhengde Qiongtai Zhi” records (6), the MY chicken breed originated in Wuzhishan City and has a history going back more than 500 years. The Wuzhishan Nanshenghuasong Ant Chicken Special Breeding Cooperative has been committed to breeding and preserving this breed since its establishment in 2011. The average weight of roosters and hens is 1.36 and 1.05 kg, respectively, which is lower than the average weight of WC (7) and BW (8).

Whole-genome resequencing is a high-throughput DNA sequencing technique that allows for the sequencing of an individual’s entire genome. Compared to traditional molecular marker and gene fragment sequencing methods, whole-genome resequencing offers higher resolution and greater coverage (9). Performing whole-genome resequencing can reveal comprehensive genomic information, including genome structure, functionality, and variation (10), thereby providing a powerful tool for the identification of new breeds. Comparing the genomic sequences of new breeds and known breeds enables the determination of their genetic relationships as well as the classification of new breeds (11). Additionally, whole-genome resequencing can help identify the genomic structure and functional features of new breeds, furthering the understanding of their biological characteristics and evolutionary history (12). New breeds must be compared with and validated against known breeds to ensure the accuracy and reliability of their identification.

In this study, to confirm that the MY chicken breed is indeed a novel genetic resource, we explored the differences between MY chickens and WC, DZ, BW, and Longsheng Feng (LF) chickens using whole-genome resequencing. Our findings may provide a basis for further exploration of the MY breed at the molecular level.

## 2 Materials and methods

### 2.1 Sample collection

**Figure 1:**
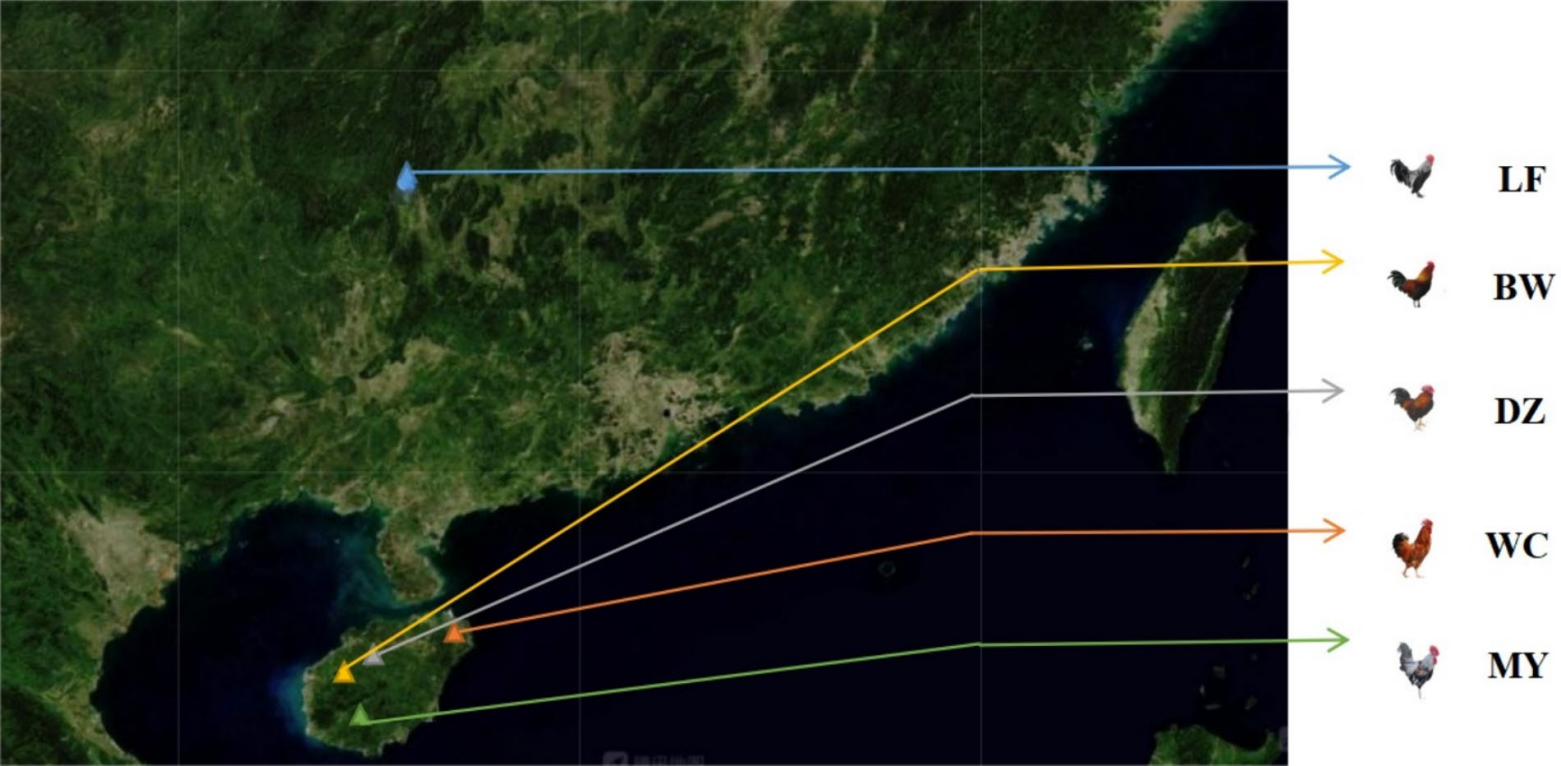
Distribution map of the five breeds. LF, Longsheng Feng chickens; BW, Bawangling chickens; DZ, Danzhou chickens; WC, Wenchang chickens; MY, Wuzhishan ant chickens.

The chickens used in this study were healthy adult birds and included 20 MY chickens from Wuzhishan Nanshenghuasong Ant Chicken Special Breeding Cooperative; 10 BW chickens from Changjiang Hongfeng Bawangling Chicken Development Co., Ltd.; 10 WC chickens from Hainan Baiguozi Wenchang Chicken Breeding Co., Ltd.; 10 DZ chickens from Hainan Rekeyuan Ecological Breeding Co., Ltd.; and 8 LF chickens from Longsheng County Hongsheng Poultry Co., Ltd. (Figure 1). Blood samples were collected from the wing vein of each chicken, mixed with acid citrate dextrose (ACD) anticoagulant, and stored at −20°C until needed.

### 2.2 DNA extraction, library construction, and sequencing

DNA was extracted from the blood samples using a blood genomic DNA extraction kit (Tiangen Biotech Co. Ltd) according to the manufacturer’s instructions. The GenoBaits DNA Library Prep Kit for ILM was used to construct resequencing libraries for qualified DNA. After library construction, Qubit 2.0 was used for preliminary quantitation, and the effective concentrations of the libraries were further verified by qPCR to ensure the quality of the libraries. After quality control, the libraries were sequenced on the Beijing Genomics Institution (BGI) MGI-2000/MGI-T7 sequencing platform in PE150 mode.

### 2.3 Quality control and alignment

Raw reads were filtered using fastp (13) (v.0.20.0, parameters: -n 10 -q 20 -u 40) to obtain clean reads. BWA-MEM (14) software was used to align the clean reads to the chicken reference genome.^1^ The HaplotypeCaller module of the GATK (15) (v.4.0.4.0) was used to detect genome-wide SNPs and insertions/deletions (InDels). To obtain SNPs with high reliability, bi-allelic loci, SNPs with a minor allele frequency (MAF) less than 0.05, those with a missing rate greater than 10%, those with a heterozygous ratio greater than 30%, and those with a detection rate lower than 90% were discarded.

### 2.4 Genetic diversity and linkage disequilibrium (LD)

MAF, polymorphic information content (PIC), observed allele number (Ao), expected allele number (Ae), observed heterozygosity (Ho), and expected heterozygosity (He) were calculated using PLINK software (16). Population nucleotide diversity (θπ) analysis refers to calculating differences in nucleotide diversity among different populations. By sliding a 100-kb window across the genome, the average π value for each window region was calculated, yielding the variation of π values in different genome regions. The fixation index (F-statistic, F_ST_) was calculated based on the SNP dataset (17) according to the method described by Weir et al. (18). The LD size between two markers was calculated using Haploview software (19). The results were processed and visualized using R script.

### 2.5 Population structure

To infer the population structure of the five chicken breeds, a principal component analysis (PCA) was performed with GCTA software (v.1.92.4) based on filtered SNP markers. Admixture software (v.1.3) was used to infer the population structure, assuming that the number of clusters (*K*-value) of the samples was 2–5, and then for clustering the samples accordingly. MEGA-X software (p-distance model with 1,000 bootstrap replicates) was used to construct a phylogenetic tree using the neighbor-joining (NJ) method based on the filtered SNP markers (20).

## 3 Results

### 3.1 Whole-genome sequencing and alignment

The statistics relating to sequencing data quality are presented in Supplementary Table S1. The number of raw reads for each sample ranged from 67,002,590 to 112,155,842, and the number of clean reads for each sample ranged from 66,987,958 to 112,120,802. A total of 598.03 Gb of clean data was obtained, averaging 10.31 Gb per bird. The average Q20 reached 97.48% and the average Q30 was 92.25%. The number of aligned reads ranged from 66,834,495 to 111,842,494, the alignment rate ranged from 99.71 to 99.84%, the average alignment rate was 99.78%, and the coverage ranged between 97.40 and 98.43% (Supplementary Table S2).

### 3.2 Screening of SNPs and InDels

**Figure 2:**
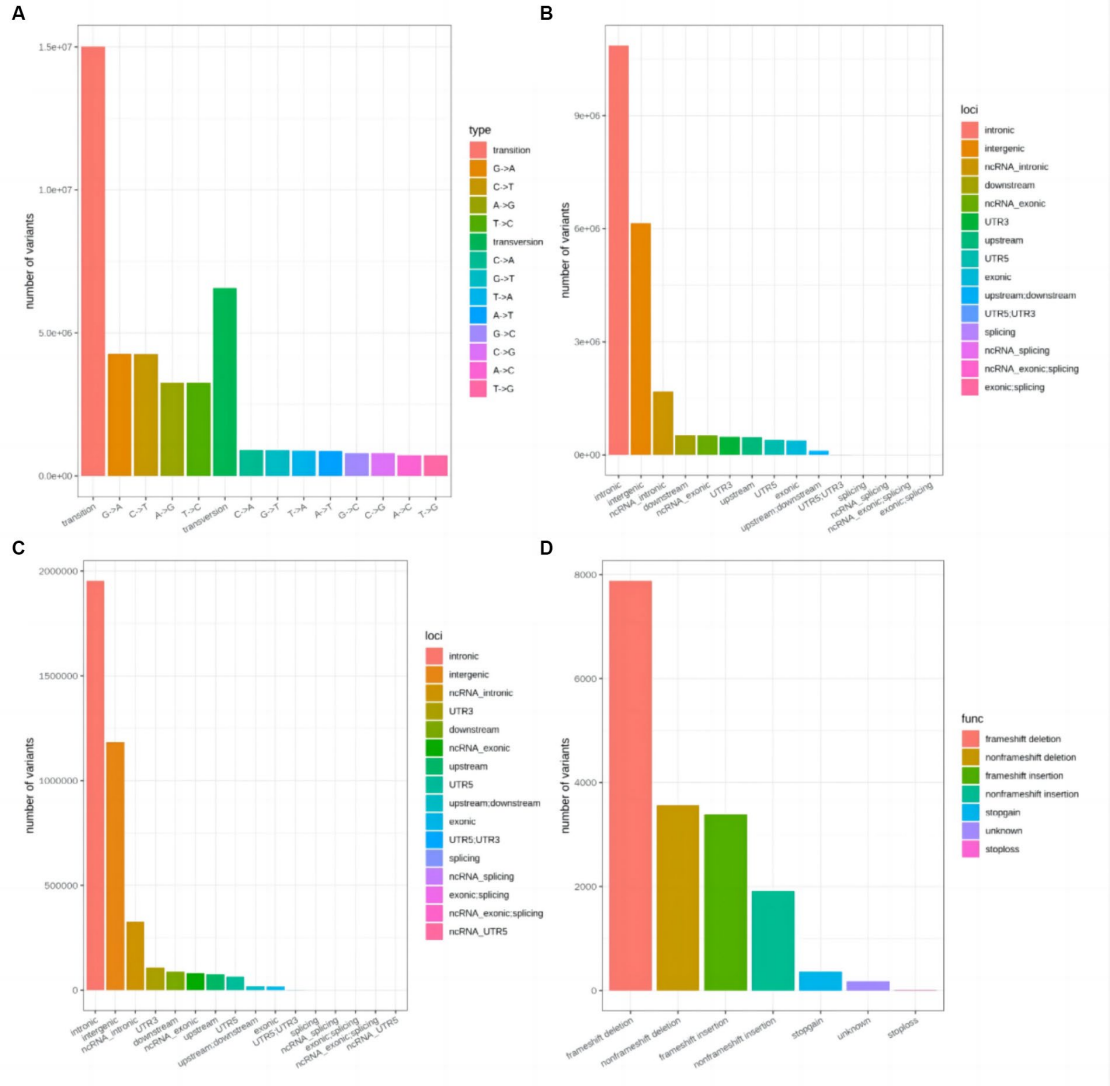
Single-nucleotide polymorphism (SNP) and insertion/deletion (InDel) screening results. **(A)** Statistical map of the SNPs. **(B)** Statistical map of SNP positions. **(C)** Statistical map of InDel positions. **(D)** Statistical map of InDel functional annotations in coding sequence (CDS) regions.

A total of 21,586,378 SNP sites were identified, including 15,014,517 transitions and 6,571,861 transversions (Figure 2A). Most SNPs were located in intronic and intergenic regions (Figure 2B). The SNPs were divided into 12 categories, with transitions accounting for 34.78% of the total. In all, 4,253,341 InDel sites were detected, including 2,174,979 deletions and 2,078,362 insertions (Figure 2C). The number of mutations located in coding sequences (CDSs) was 17,272, with frameshift deletions (7,880) accounting for 45.6% of these (Figure 2D).

### 3.3 Genetic diversity and LD

MAF, PIC, Ao, Ae, Ho, and He were calculated to evaluate the genetic diversity among the five breeds. The genetic diversity parameters are shown in Supplementary Table S3. MAFs ranged from 0.1220 to 0.1382, with an average of 0.1297. PICs ranged from 0.1418 to 0.1615, with the average being 0.1486. Ao ranged from 1.5541 to 1.8157, averaging 1.6587. Ae ranged between 1.2792 and 1.3167, with an average of 1.2948. Ho ranged from 0.1389 to 0.1657, with an average of 0.1581. He ranged from 0.1735 to 0.1957, with an average of 0.1807. Chickens of the MY breed had the highest MAF (0.1382), PIC (0.1615), Ho (0.1657), and He (0.1957) values.

**Figure 3:**
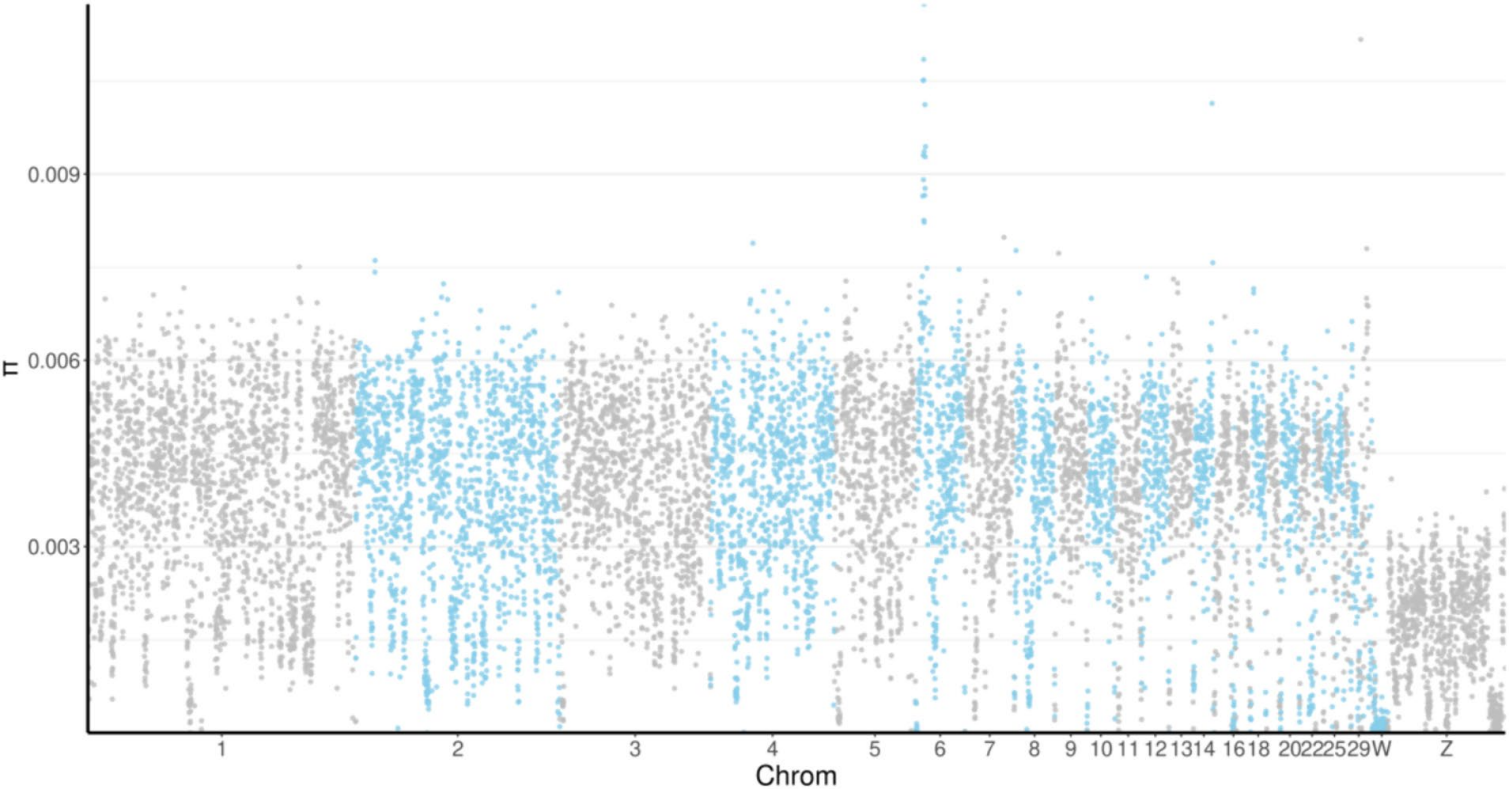
Manhattan plot of nucleotide diversity (π) distribution. The horizontal axis indicates the position of the genome, and the vertical coordinate indicates the π value of the windows. Chrom, chromosome.

Nucleotide diversity (θπ) analysis refers to the calculation of nucleotide diversity differences among different breeds and can reflect the degree of genetic diversity of a breed. The π value for the MY breed was 0.003468572 and was only lower than that of the WC breed. We found that the π values were highest on chromosome 6 and lowest on chromosome Z in all the breeds (Figure 3).

The F_ST_ is a measure of population differentiation and genetic distance and indicates the degree of genetic differentiation between populations. The larger the F_ST_ value, the greater the genetic difference. The F_ST_ estimation results are shown in Supplementary Table S4. The F_ST_ values among all the groups ranged from 0.05 to 0.15, indicating that there was a moderate degree of genetic differentiation between the populations. The lowest F_ST_ value (0.055585709) was detected between the MY and WC breeds, while the highest (0.086456908) was found between the MY and LF breeds.

**Figure 4:**
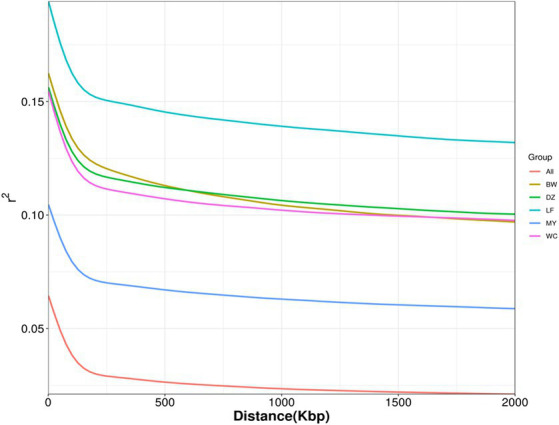
Linkage disequilibrium decay plot. LF, Longsheng Feng chickens; BW, Bawangling chickens; DZ, Danzhou chickens; WC, Wenchang chickens; MY, Wuzhishan ant chickens.

LD refers to a non-random association between alleles at different loci within a population; that is, two genes will exhibit some degree of linkage as long as they are not inherited completely independently. LD decay analysis showed that the MY breed had the highest decay rate, followed by the BW, DZ, and LF breeds (Figure 4). After 20 kb, the LD coefficient (*r*^2^) of MY chickens was lower than 0.1. After 120 kb, the decline in LD coefficients slowed down in all the breeds.

### 3.4 Population structure

**Figure 5:**
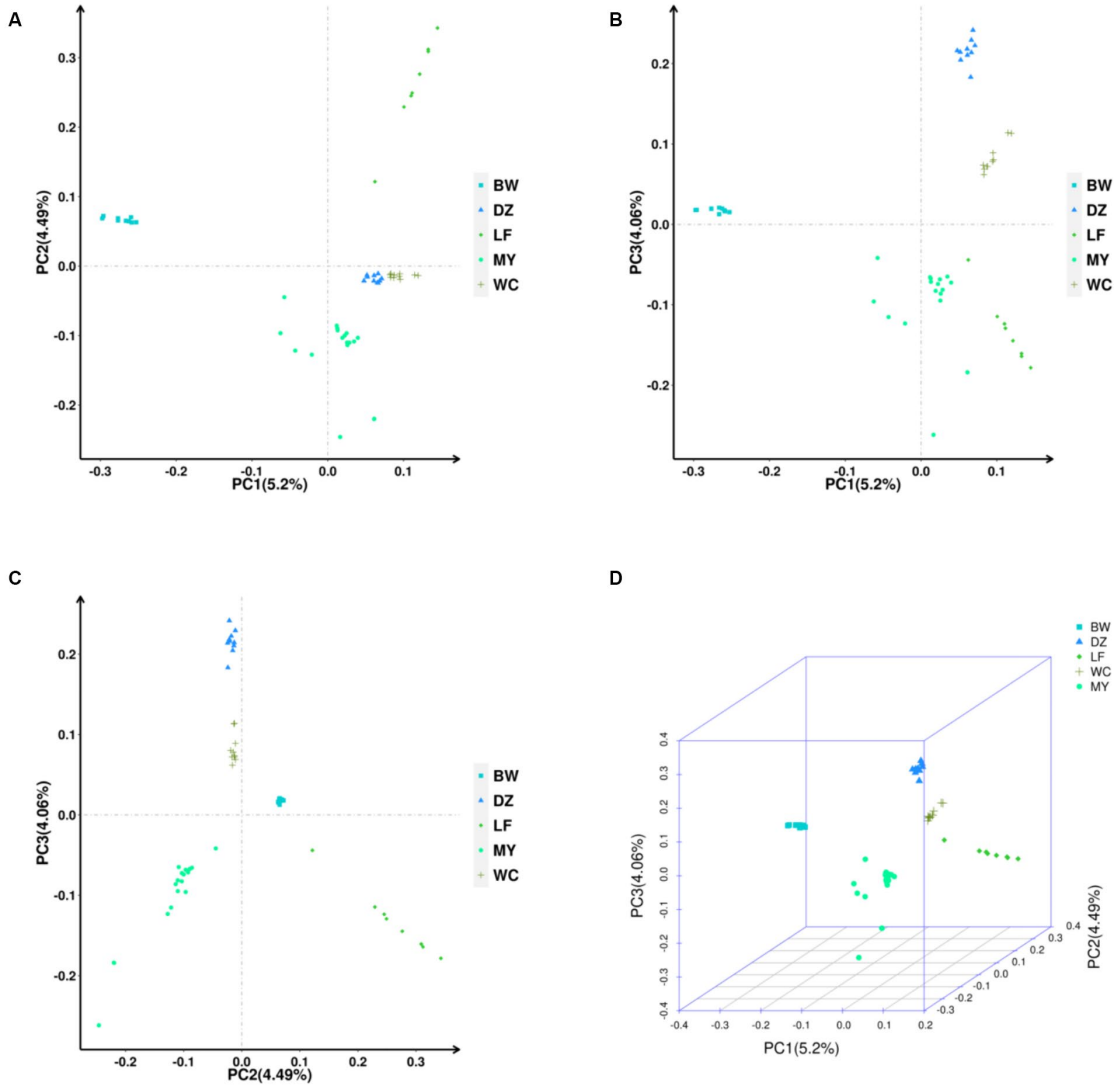
Principal component analysis of the five chicken breeds. **(A)** PC1/PC2. **(B)** PC1/PC3. **(C)** PC2/PC3. **(D)** 3D PCA Scatter Plot. LF, Longsheng Feng chickens; BW, Bawangling chickens; DZ, Danzhou chickens; WC, Wenchang chickens; MY, Wuzhishan ant chickens.

The PCA showed that the contribution of the top three principal components—PC1, PC2, and PC3—was 5.20, 4.49, and 4.06%, respectively. As shown in Figure 5A, the MY, BW, and LF breeds clustered separately, while the DZ and WC breeds clustered together in one branch. Figures 5B,C show that the MY and the other four chicken breeds were clustered separately in one branch. As can also be seen from the 3D plot in Figure 5D, the MY population is clearly distinguishable from the other breeds These results indicated that there were significant differences in population structure between the MY and the other four chicken breeds.

**Figure 6:**
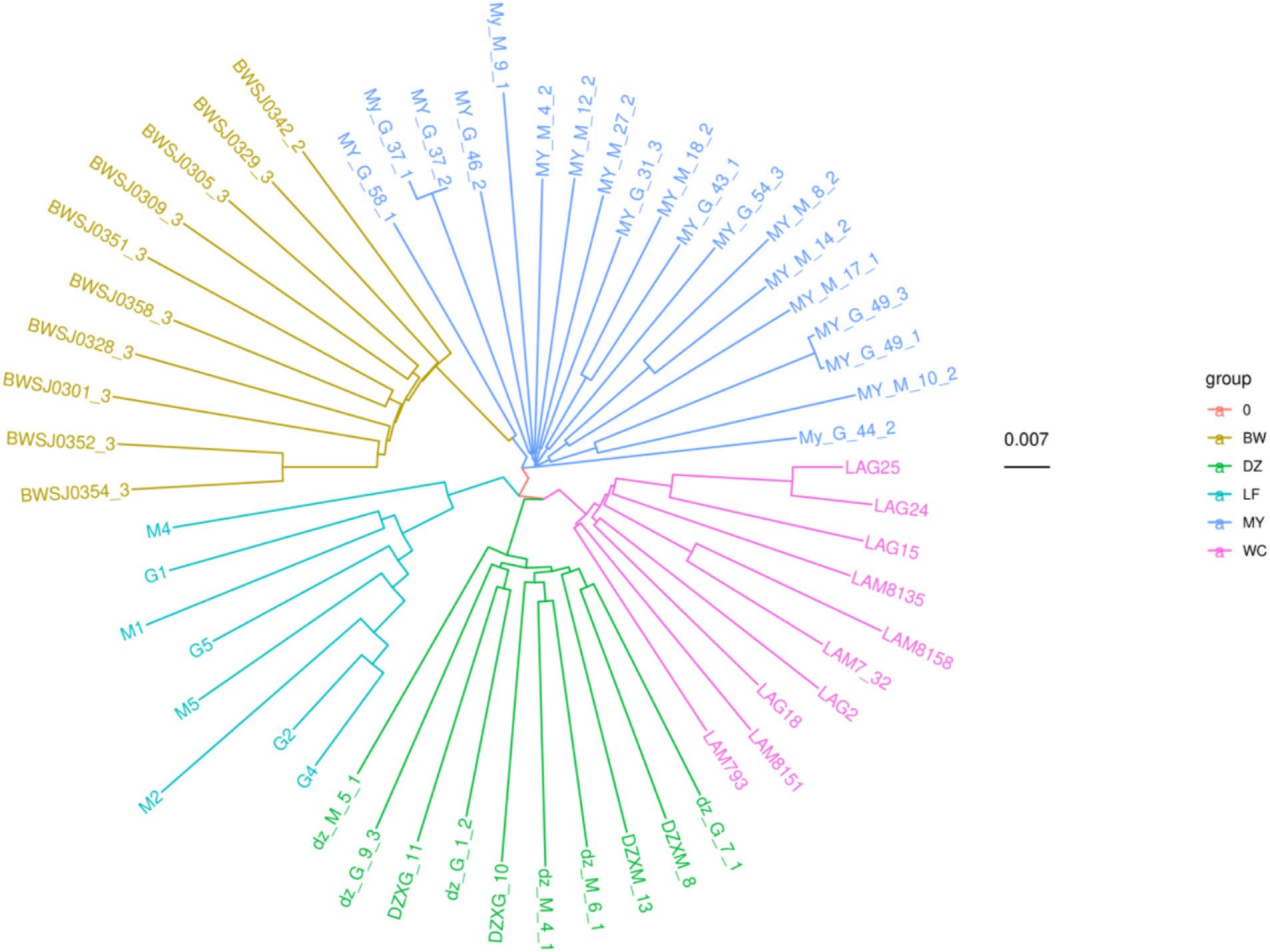
Phylogenetic tree of five chicken breeds. The “group” label indicates the different chicken breeds. The number 0.007 indicates that the branch of this length represents a genetic variation of 0.007 in the genome.

The results of the phylogenetic tree reconstruction using the NJ method are shown in Figure 6. All the birds from individual indigenous chicken breeds clustered together with birds of their respective breeds, forming five distinct branches. This suggested that the five breeds were independent from each other. Additionally, the genetic relationship between the MY and BW breeds was found to be relatively close, consistent with the PCA results.

**Figure 7:**
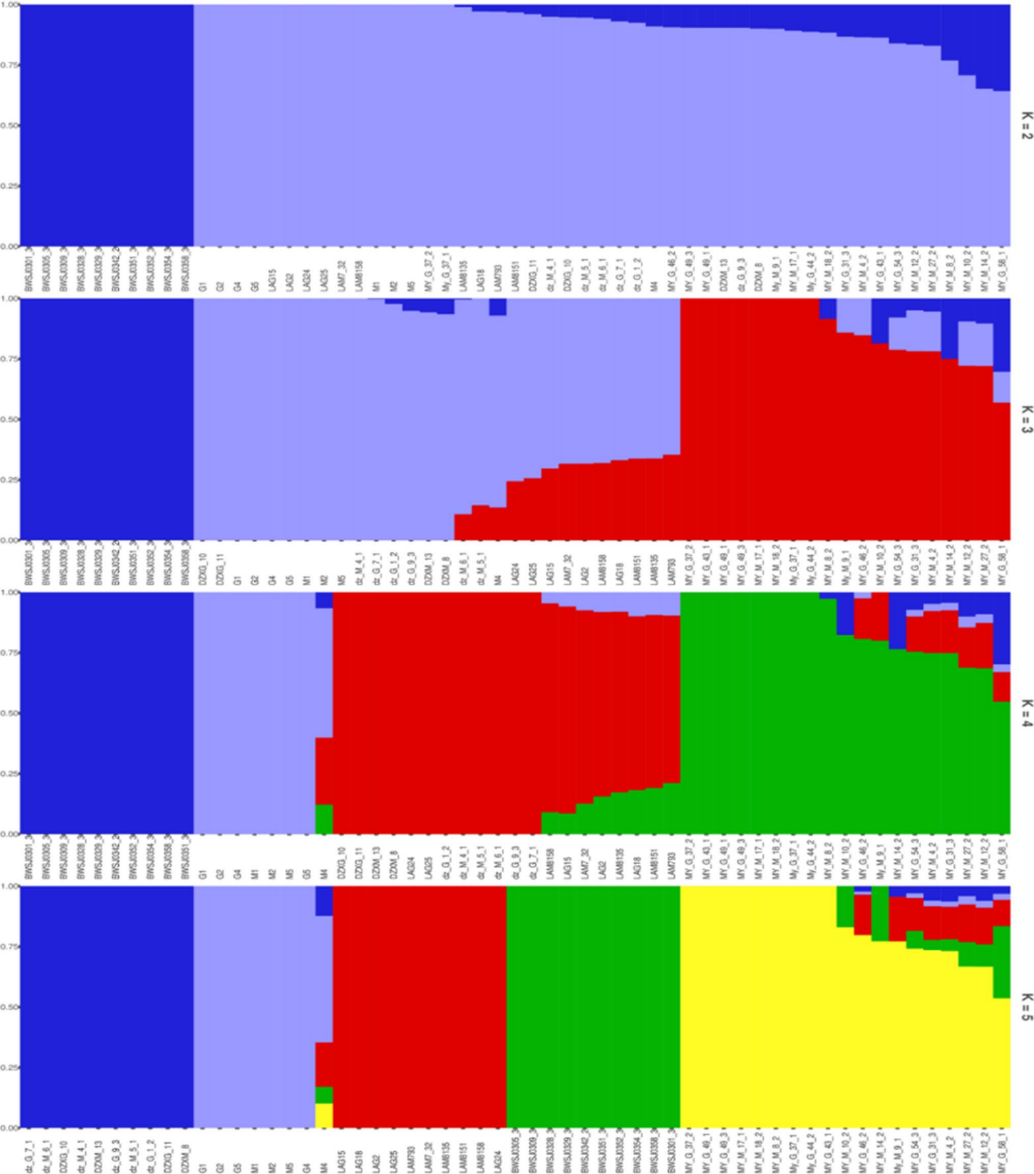
Histogram of the genetic composition of the samples. Each color represents a cluster within each *K* value.

The genetic composition of each sample in each subgroup was plotted as a bar chart using the pophelper R package (v.2.2.7) (Figure 7). Analysis of the genetic bar chart demonstrated that when *K* = 2, birds of the BW breed clustered separately, those of the LF and WC breeds clustered together, and those of the MY and DZ breeds had mixed ancestry. When *K* = 3, birds of the BW, LF, and MY breeds were clustered separately, with some birds of the MY breed having mixed ancestry from the first two groups. When *K* = 4, animals of the BW, LF, DZ, and MY breeds were clustered separately, with some birds of the MY breed having mixed ancestry from the first three groups. When *K* = 5, each chicken breed was clustered separately, but again, some MY birds shared ancestry with the other four chicken breeds.

## 4 Discussion

The identification of genetic resources of new varieties should include comparisons at both the phenotypic and molecular levels (21). Genome resequencing technology is widely used for the detection of genetic variation in large breeds, given its greater coverage, accuracy, and efficiency compared with traditionally used methods (22–24). In this study, whole-genome resequencing was used for the validation of the MY breed as a novel genetic resource.

Generally, the greater the quality of sequencing data and the higher the alignment rate, the more reliable the subsequent results (25). In this study, the average Q20 value of our data was 97.48%, and the average Q30 value was 92.25%. Additionally, the average alignment rate was 99.78%. Our Q20 or Q30 rates were substantially higher than those reported by Shuli et al. (26) and Wei et al. (27), while the alignment rate of our data was higher than that achieved by Yin et al. (28) and Zhou et al. (29). This indicated that our sequencing data were of sufficient quality for use in subsequent analysis.

The identification and evaluation of a new resource entails determining its genetic background and classification and assessing its genetic diversity and genetic structure, thus providing a basis for the genetic improvement of that resource (30). In this study, the MAF, PIC, Ho, and He were higher in birds of the MY breed than in those of the other four breeds, indicating that the MY breed was different from the other four breeds. Ho and He of MY are lower than those reported by Tian et al. (31). The reason may be that the sample size is insufficient, leading to the low genetic diversity index of Ho and He. Importantly, the MY breed has not been commercially exploited and has not been subjected to significant artificial intervention, which leads to high genetic diversity.

Nucleotide diversity (θπ) reflects the degree of genetic diversity of a breed (32). The lower the polymorphism, the higher the degree of selection, and the lower its genetic diversity. In this study, we found that MY chickens had the second highest π value among the five breeds. This may reflect that most MY chickens that are reared in the deep mountain forests of the southern tropical region of Hainan Island have undergone limited artificial selection, which likely increased their genetic diversity. Additionally, for all the breeds tested, the π values were highest on chromosome 6 and lowest on chromosome Z, indicating that there was a higher degree of correlation between artificial selection traits and chromosome 6 than between artificial selection traits and chromosome Z. Further research will help confirm these findings and identify the specific genetic mechanisms associated with artificial selection in the MY breed.

LD is widely used in association analysis and genetic parameter estimation. In general, an LD coefficient greater than 0.8 is indicative of a strong correlation between two loci. Meanwhile, an LD coefficient lower than 0.1 is indicative of a lack of correlation, which occurs when there is a long distance between two loci or when both loci have a high mutation rate, which reduces the degree of association between them (33). In this study, we found that MY chickens had the lowest LD coefficient among the five breeds studied, while LF chickens had the highest. These results reflect that MY birds were subjected to the lowest degree of artificial selection and LF birds the highest. The average LD coefficient of the MY breed was less than 0.1, while those of the three other Hainan breeds—WC, BW, and DZ—were all higher than 0.1 and were similar to each other (0.105110967, 0.108442684, and 0.109120896, respectively). This indicated that MY birds underwent almost no artificial selection, while the other three Hainan breeds experienced a similar degree of artificial selection. The results of the LD analysis were consistent with those of genetic diversity analysis.

The F_ST_ is a measure of population differentiation and genetic distance; the greater the F_ST_, the greater the differences between populations (34). In this study, the F_ST_ values of the five chicken breeds ranged from 0.05 to 0.15, which reflects a moderate level of genetic diversity in chickens (35). When the MY breed was compared to the other four breeds, the largest F_ST_ value was found between the MY and LF breeds, followed by the BW, DZ, and WC breeds, which was also correlated with their geographical locations. The MY breed is distributed in the southern tropical region of Hainan Island, the southernmost province of China (5). The DZ, WC, and BW breeds are distributed in the northern subtropical region of Hainan Island (7, 8), and the LF breed is found in Longsheng County, Guangxi Province (36). This result suggested that the MY breed differs from the other four breeds in terms of population differentiation and genetic distance. The F_ST_ values were higher for the Z chromosome than for the other chromosomes, which was consistent with the results of Li et al. (37). Investigating the level of genomic variation in the sex chromosome and the autosomes may help reveal the role of sexual selection in the long-term evolution of a species (38).

Many studies have used whole-genome sequencing to compare the structure of livestock and poultry genetic resources and explore the genetic relationships among them (39–41). In this study, the five indigenous chicken breeds were clustered into five different branches on the phylogenetic tree. The MY breed was found to be located on a separate branch, forming a distinct cluster from the other four breeds. This suggested that the MY breed has unique genetic characteristics, supporting its classification as a new breed. This finding was consistent with the results of the PCA as well as with the geographical distribution of the breeds. When *K* = 3–5, the MY breed could clearly be distinguished from other breeds, and some birds of the MY breed shared a common ancestry with three local Hainan chicken breeds. This is likely due to the genetic exchange resulting from their coexistence on Hainan Island. These results indicated that the MY breed is distinct from the other four indigenous breeds regarding population structure.

In summary, in this study, to verify that the MY breed is indeed a new genetic resource, we compared genomic genetic diversity, nucleotide diversity, the fixation index, the LD coefficient, and the phylogenetic tree relationship between the MY breed and four other chicken breeds via whole-genome resequencing. The MY breed had the second highest genomic genetic diversity and nucleotide diversity and the lowest LD coefficient among the five breeds. In addition, the phylogenetic tree analysis showed that individual birds of the MY breed were clustered into one group separate from the other breeds. Taken together, the results of this study showed that the MY breed is completely different from the other four breeds and can be considered a new genetic resource.

## Data availability statement

The data presented in the study are deposited in the Sequence Read Archive (SRA) repository, accession number PRJNA943094. The data can be found on https://www.ncbi.nlm.nih.gov/bioproject/?term=PRJNA943094.

## Ethics statement

The animal study was approved by Institute of Animal Science & Veterinary Medicine, Hainan Academy of Agricultural Sciences. The study was conducted in accordance with the local legislation and institutional requirements.

## Author contributions

LG: Project administration, Supervision, Writing – original draft. FW: Writing – original draft. XinZ: Methodology, Writing – review & editing. XiaZ: Methodology, Writing – review & editing. YaC: Funding acquisition, Writing – review & editing. LL: Validation, Writing – review & editing. XL: Funding acquisition, Writing – review & editing. SM: Funding acquisition, Writing – review & editing. ZC: Investigation, Writing – review & editing. ZH: Investigation, Writing – review & editing. YS: Data curation, Writing – review & editing. DW: Resources, Writing – review & editing. SW: Resources, Writing – review & editing. YoC: Funding acquisition, Project administration, Supervision, Writing – review & editing. TX: Investigation, Supervision, Writing – review & editing.

## Footnotes

^1^ https://ftp.ncbi.nlm.nih.gov/genomes/all/GCF/016/699/485/GCF_016699485.2_bGalGal1.mat.broiler.GRCg7b/GCF_016699485.2_bGalGal1.mat.broiler.GRCg7b_genomic.fna.gz

## References

[1] China National Commission of Animal Genetic Resources. Animal genetic resources in China Poultrys [M]. Beijing: China Agriculture Press (2011).

[2] Mcmahon BJ Teeling EC Höglund J. How and why should we implement genomics into conservation? [J]. Evol Appl. (2014) 7:999–1007. doi: 10.1111/eva.12193, PMID: 25553063 PMC4231591

[3] Lopes MS MendonÃ§a D Rojer H Cabral VÃ3 Bettencourt SÃX da CÃ¢mara Machado A. Morphological and genetic characterization of an emerging Azorean horse breed: the Terceira pony [J]. Front Genet. (2015) 6:62. doi: 10.3389/fgene.2015.00062 25774165 PMC4343030

[4] Andersson L Georges M. Domestic-animal genomics: deciphering the genetics of complex traits [J]. Nat Rev Genet. (2004) 5:202–12. doi: 10.1038/nrg1294, PMID: 14970822

[5] Gu LH. Performance study of Wuzhishan ant chicken germplasm [J]. Chin Livest Poult Breed. (2022) 18:5–7+3.

[6] Zhou T. Zhengde Qiongtai Zhi [M]. Qiongzhou Prefecture: Hainan Press (2006).

[7] Dazhuang Z An F Lihong G. Current situation analysis of Wenchang chicken industry [J]. Guide Chin Poult. (2022) 39:18–21.

[8] Fei W Xiaochun L Kebang W. Growth curve fitting and correlation analysis between body weight and body size in Bawangling chicken [J]. J South Agric. (2014) 45:870–4. doi: 10.3969/j:issn.2095-1191.2014.5.870

[9] Kim JY Hwang JE Eo SH Kang SG Moon JC Kim JA. Development of InDel markers for interspecific hybridization between hill pigeons and feral pigeons based on whole-genome re-sequencing [J]. Sci Rep. (2022) 12:22618. doi: 10.1038/s41598-022-27147-1, PMID: 36585442 PMC9803650

[10] Martin J Schackwitz W Lipzen A. Genomic sequence variation analysis by resequencing. Methods Mol Biol. (2018) 1775:229–39. doi: 10.1007/978-1-4939-7804-5_18 29876821

[11] Liu M Yu C Zhang Z Song M Sun X Piálek J. Whole-genome sequencing reveals the genetic mechanisms of domestication in classical inbred mice [J]. Genome Biol. (2022) 23:203. doi: 10.1186/s13059-022-02772-1, PMID: 36163035 PMC9511766

[12] Fan Z Zhao G Li P Osada N Xing J Yi Y. Whole-genome sequencing of tibetan macaque (*Macaca Thibetana*) provides new insight into the macaque evolutionary history [J]. Mol Biol Evol. (2014) 31:1475–89. doi: 10.1093/molbev/msu104, PMID: 24648498 PMC4032132

[13] Chen S Zhou Y Chen Y Gu J. Fastp: an ultra-fast all-in-one Fastq preprocessor [J]. Bioinformatics. (2018) 34:i884–90. doi: 10.1093/bioinformatics/bty560, PMID: 30423086 PMC6129281

[14] Li H Durbin R. Fast and accurate short read alignment with burrows-wheeler transform [J]. Bioinformatics. (2009) 25:1754–60. doi: 10.1093/bioinformatics/btp324, PMID: 19451168 PMC2705234

[15] Garimella K Altshuler D Gabriel S Daly M DePristo MA. The genome analysis toolkit: a MapReduce framework for analyzing next-generation Dna sequencing data [J]. Genome Res. (2010) 20:1297–303. doi: 10.1101/gr.107524.110, PMID: 20644199 PMC2928508

[16] Purcell S Neale B Todd-Brown K Thomas L Ferreira MAR Bender D. Plink: a tool set for whole-genome association and population-based linkage analyses [J]. Am J Hum Genet. (2007) 81:559–75. doi: 10.1086/519795, PMID: 17701901 PMC1950838

[17] Balloux F Lugon-Moulin N. The estimation of population differentiation with microsatellite markers [J]. Mol Ecol. (2002) 11:155–65. doi: 10.1046/j.0962-1083.2001.01436.x 11856418

[18] Weir BS Cockerham CC. Estimating F-statistics for the analysis of population structure [J]. Evolution. (1984) 38:1358–70. doi: 10.1111/j.1558-5646.1984.tb05657.x, PMID: 28563791

[19] Barrett JC Fry B Maller J Daly MJ. Haploview: analysis and visualization of Ld and haplotype maps [J]. Bioinformatics. (2005) 21:263–5. doi: 10.1093/bioinformatics/bth457, PMID: 15297300

[20] Tamura K Stecher G Peterson D Filipski A Kumar S. Mega6: molecular evolutionary genetics analysis version 6.0 [J]. Mol Biol Evol. (2013) 30:2725–9. doi: 10.1093/molbev/mst197 24132122 PMC3840312

[21] Gálová Z Urminská D. The differences between the old and new barley varieties on the basis of Hordein polymorphism with respect to qualitative parameters [J]. J Microbiol Biotechnol Food Sci. (2015) 4:108–110. doi: 10.15414/jmbfs.2015.4.special2

[22] Cao T Zhou X Zhao YH. Progress in the application of whole-genome sequencing in livestock and poultry [J]. China Anim Husb Vet Med. (2021) 48:3403–14. doi: 10.16431/j:cnki.1671-7236.2021.09.032

[23] Chen S Man C Du L Zhang W Wang F. Whole-genome sequencing reveals the genomic characteristics and selection signatures of Hainan black goat [J]. Genes (Basel). (2022) 13:1539. doi: 10.3390/genes13091539 36140707 PMC9498695

[24] Xiong X Liu J Rao Y. Whole genome resequencing helps study important traits in chickens [J]. Genes (Basel). (2023) 14:1198. doi: 10.3390/genes14061198, PMID: 37372379 PMC10298700

[25] Wang XL Wu YJ Ba G. Analysis of genetic diversity and population structure of Changdu black sheep based on re-sequencing data [J]. J Domest Anim Ecol. (2022) 43:30–5. doi: 10.3969/j.issn.1673-1182.2022.10.005

[26] Xue Q Han W Li GH. Genetic evolution of Tianjin monkey chicken based on simplified genome sequencing [J]. China Poult. (2022) 44:1–5. doi: 10.16372/j.issn.1004-6364.2022.07.001

[27] Shen YM Su YJ Dou X Wang KH Zou JM. Genetic evolution of 19 native chicken breeds based on rad-seq simplified genome sequencing [J]. Acta Vet Zootechnca Sin. (2020) 51:670–8. doi: 10.11843/j.issn.0366-6964.2020.04.003

[28] Yin H Li D Wang Y Zhu Q. Whole-genome resequencing analysis of Pengxian yellow chicken to identify genome-wide SNPs and signatures of selection. 3 Biotech. (2019) 9:383. doi: 10.1007/s13205-019-1902-6 PMC6778165 31656721

[29] Zhou J Chang Y Li J Bao H Wu C. Integrating whole-genome resequencing and Rna sequencing data reveals selective sweeps and differentially expressed genes related to nervous system changes in Luxi gamecocks [J]. Genes (Basel). (2023) 14:584. doi: 10.3390/genes14030584, PMID: 36980855 PMC10048732

[30] Sharma R Ahlawat S Sharma H Prakash V Gupta S Khatak S. Identification of a new Indian camel germplasm by microsatellite markers based genetic diversity and population structure of three camel populations [J]. Saudi J Biol Sci. (2020) 27:1699–709. doi: 10.1016/j.sjbs.2020.04.046, PMID: 32565685 PMC7296511

[31] Tian S Li W Zhong Z Wang F Xiao Q. Genome-wide re-sequencing data reveals the genetic diversity and population structure of Wenchang chicken in China [J]. Anim Genet. (2023) 54:328–37. doi: 10.1111/age.13293, PMID: 36639920

[32] Jai XX Ge QL Lu JX Wang Y Gao YS. Analysis of genetic diversity and origin evolution of Gushi chicken based on mitochondrial control region [J]. J Yangzhou Univ. (2022) 43:56–61.

[33] Slatkin M. Linkage disequilibrium — understanding the evolutionary past and mapping the medical future. Nat Rev Genet. (2008) 9:477–85. doi: 10.1038/nrg2361, PMID: 18427557 PMC5124487

[34] Takezaki N Nei M. Genetic distances and reconstruction of phylogenetic trees from microsatellite Dna [J]. Genetics. (1996) 144:389–99. doi: 10.1093/genetics/144.1.389, PMID: 8878702 PMC1207511

[35] Meirmans PG Hedrick PW. Assessing population structure: F(St) and related measures [J]. Mol Ecol Resour. (2011) 11:5–18. doi: 10.1111/j.1755-0998.2010.02927.x, PMID: 21429096

[36] Gu J Li S. Complete mitochondrial genome of the Longsheng Feng chicken (*Gallus gallus*) [J]. Mitochondrial DNA B Resour. (2020) 5:2911–2. doi: 10.1080/23802359.2020.1791753, PMID: 33457999 PMC7781991

[37] Li D Li Y Li M Che T Tian S Chen B. Population genomics identifies patterns of genetic diversity and selection in chicken [J]. BMC Genomics. (2019) 20:263. doi: 10.1186/s12864-019-5622-4, PMID: 30940068 PMC6446315

[38] Corl A Ellegren H. The genomic signature of sexual selection in the genetic diversity of the sex chromosomes and autosomes [J]. Evolution. (2012) 66:2138–49. doi: 10.1111/j.1558-5646.2012.01586.x, PMID: 22759291

[39] Wang Tongmiao GQ Hao B. Population structure analysis of different duck genetic resources based on whole genome resequencing [J]. Chin J Anim Sci. (2021) 57:78–81. doi: 10.19556/j.0258-7033.20210126-05

[40] Xiao Q. Analysis of the conservation status of different breeding populations of Wenchang chicken based on whole genome resequencing data [J]. Heilongjiang Anim Husb Vet Med. (2023) 13:51-4+62+134.

[41] Sun J Chen T Zhu M Wang R Huang Y Wei Q. Whole-genome sequencing revealed genetic diversity and selection of Guangxi indigenous chickens [J]. PLoS One. (2022) 17:e0250392. doi: 10.1371/journal.pone.0250392, PMID: 35290380 PMC8923445

