# Supplementary figures and images for: Molecular genetic identification of Wuzhishan ant chicken, a newly discovered resource in China
Source: Front Vet Sci. 2024 Jun 19;11:1319854. doi: 10.3389/fvets.2024.1319854 (PMC11221338; doi:10.3389/fvets.2024.1319854)

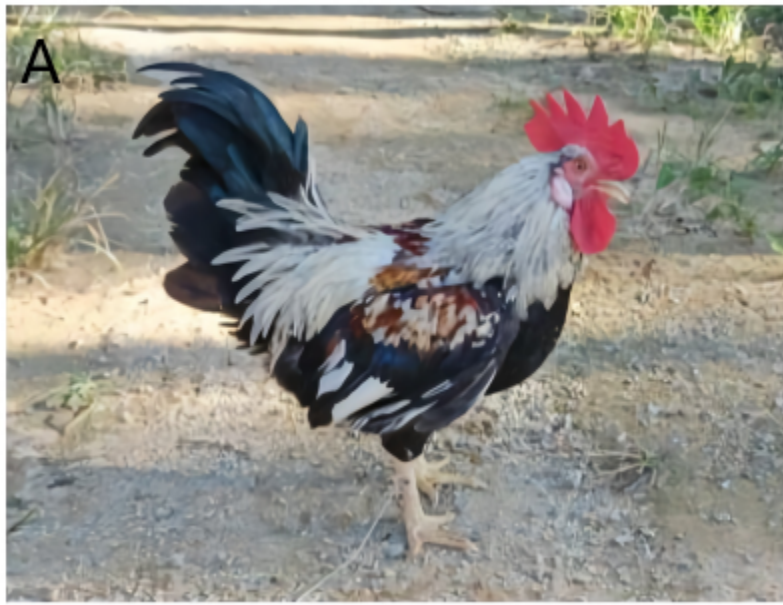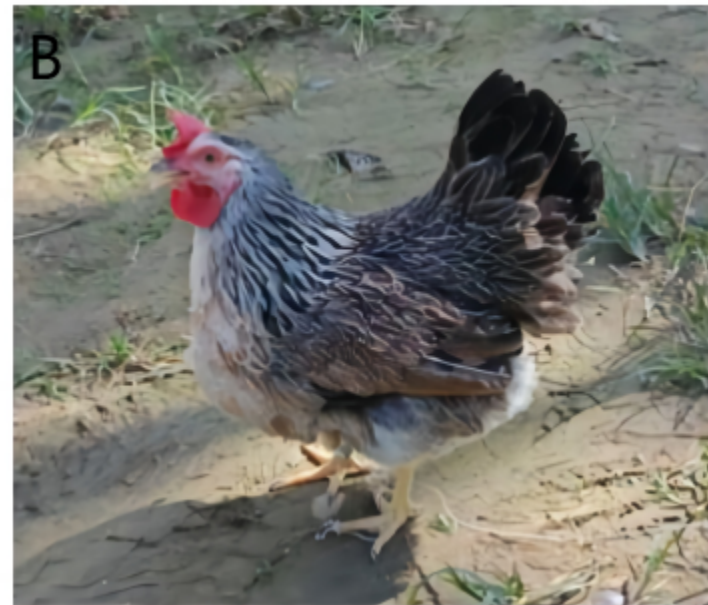

Supplementary Figure 1. Appearance of Wuzhi shan Ant Chicken (A)rooster (B)hen

Supplement: Supplementary file 1 [file Data_Sheet_1.PDF]
